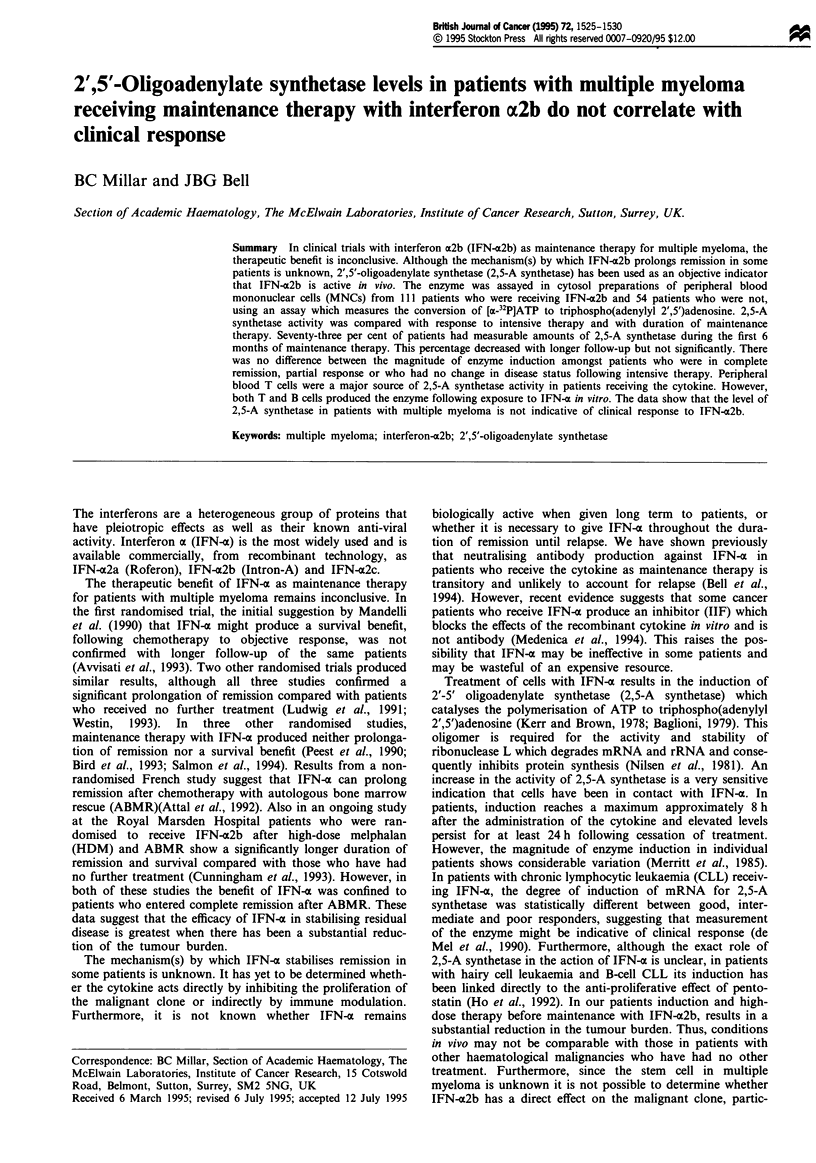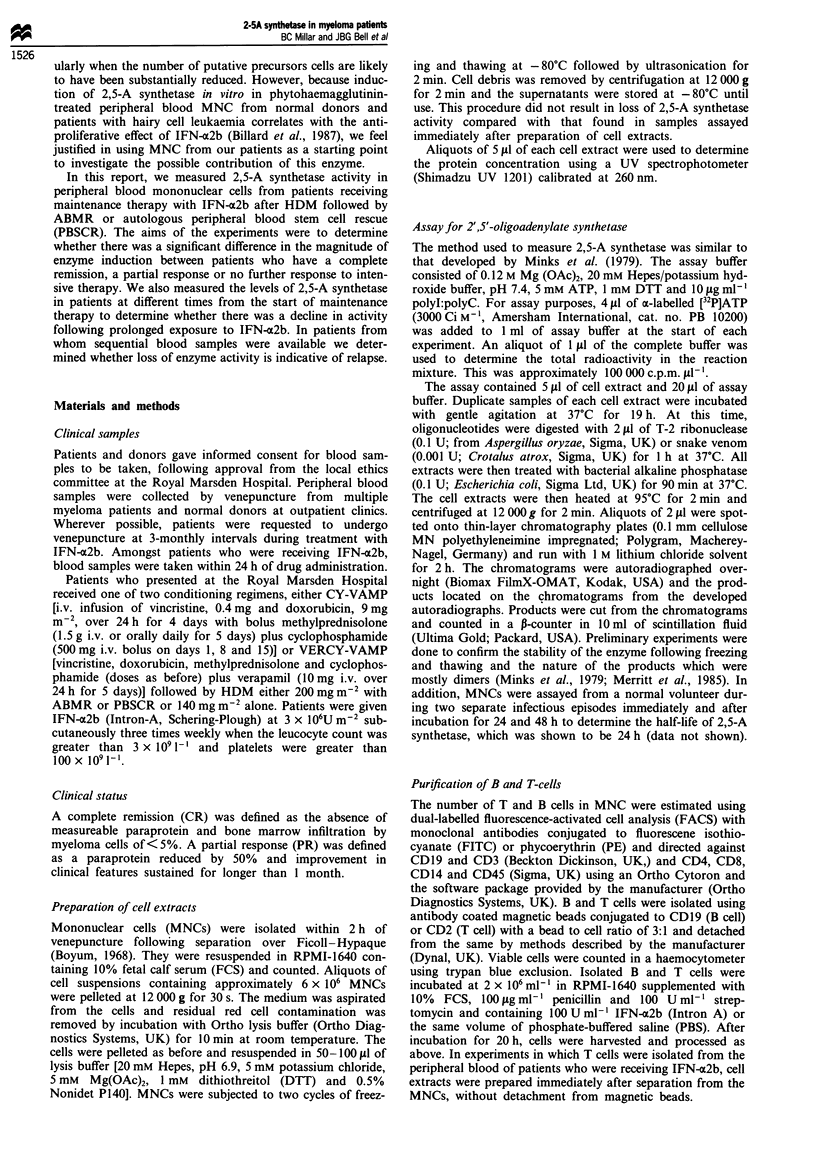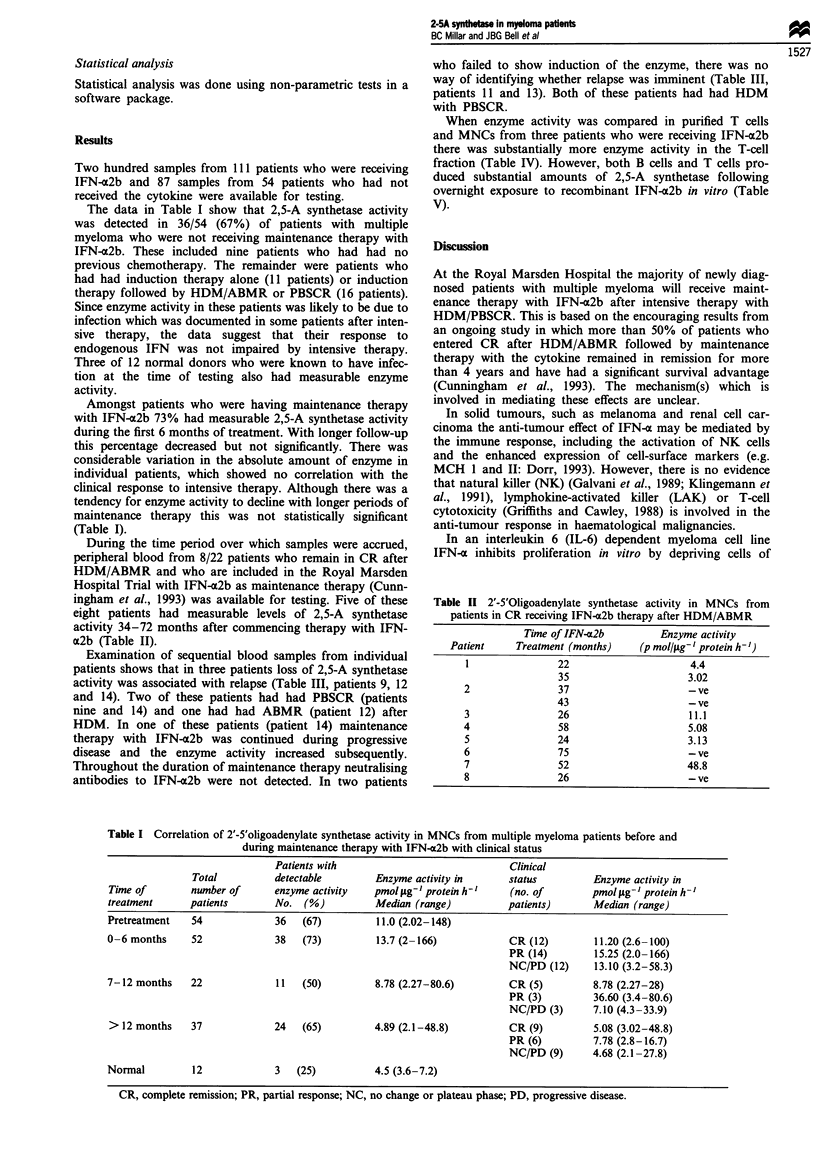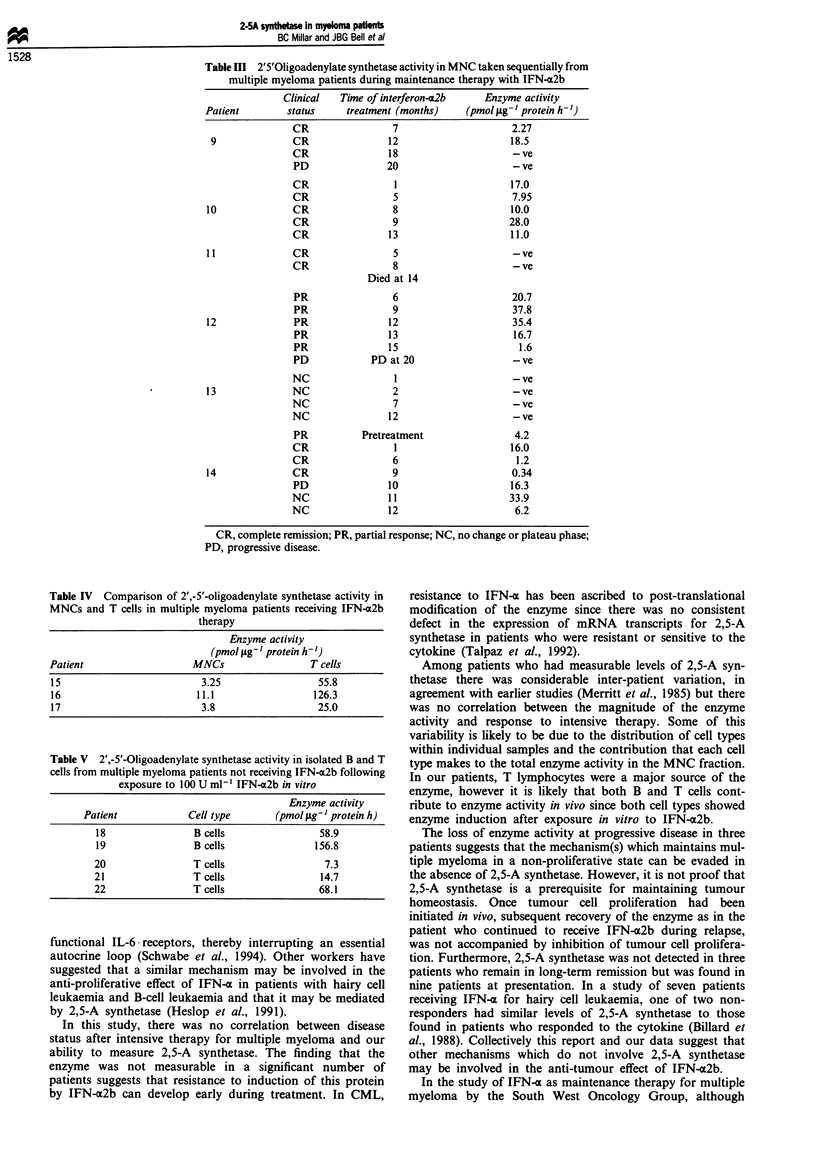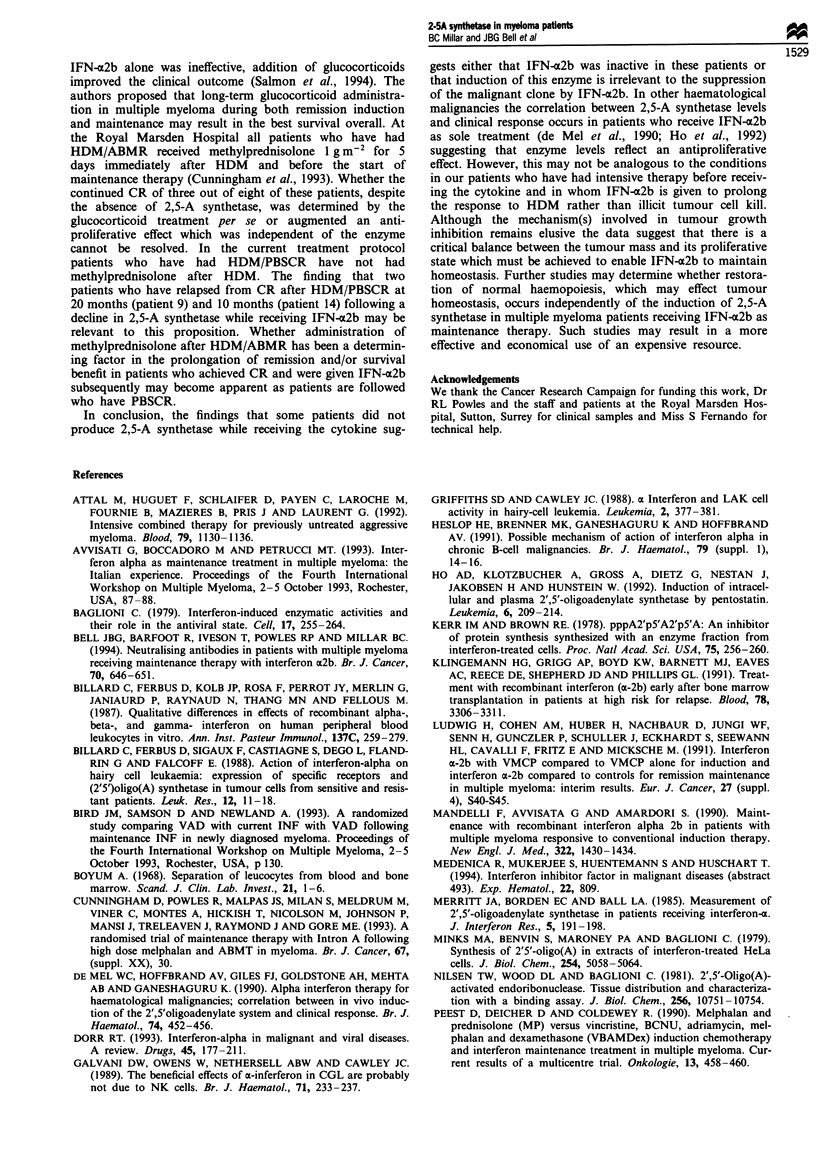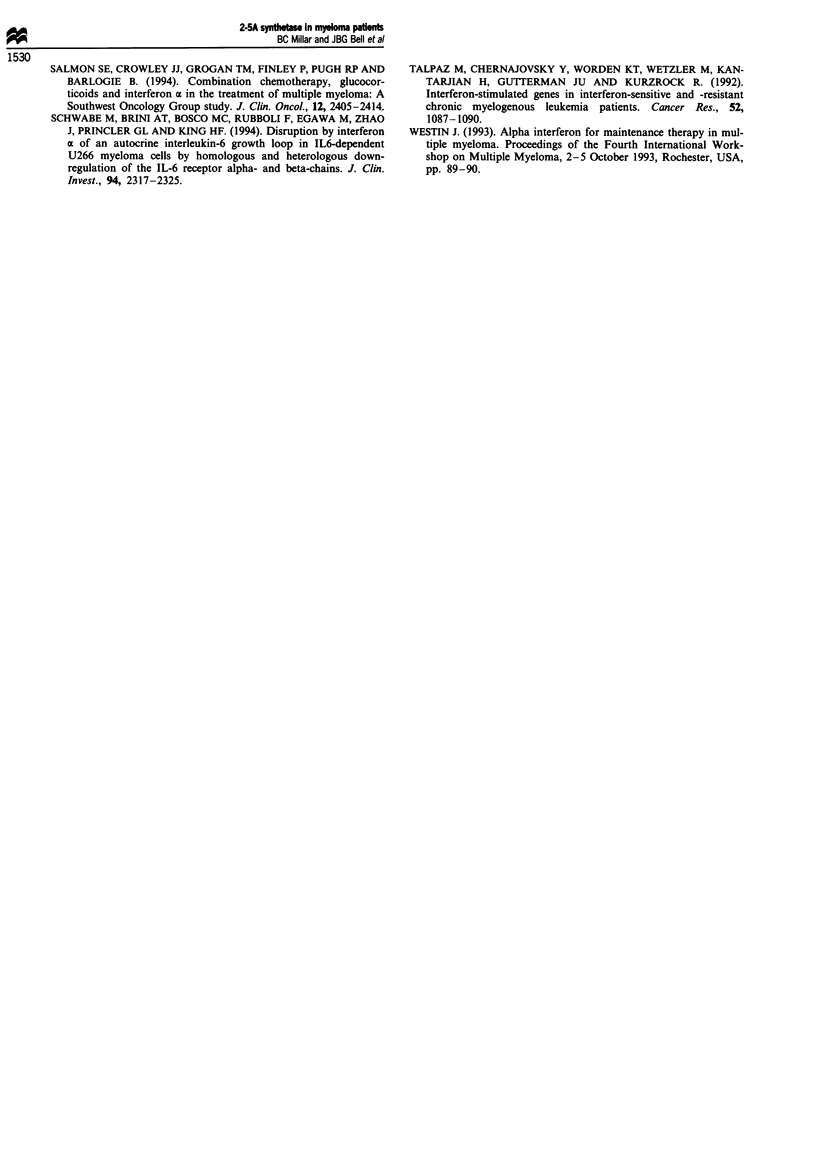# 2',5'-Oligoadenylate synthetase levels in patients with multiple myeloma receiving maintenance therapy with interferon alpha 2b do not correlate with clinical response.

**DOI:** 10.1038/bjc.1995.541

**Published:** 1995-12

**Authors:** B. C. Millar, J. B. Bell

**Affiliations:** Section of Academic Haematology, McElwain Laboratories, Institute of Cancer Research, Sutton, Surrey, UK.

## Abstract

In clinical trials with interferon alpha 2b (IFN-alpha 2b) as maintenance therapy for multiple myeloma, the therapeutic benefit is inconclusive. Although the mechanism(s) by which IFN-alpha 2b prolongs remission in some patients is unknown, 2',5'-oligoadenylate synthetase (2,5-A synthetase) has been used as an objective indicator that IFN-alpha 2b is active in vivo. The enzyme was assayed in cytosol preparations of peripheral blood mononuclear cells (MNCs) from 111 patients who were receiving IFN-alpha 2b and 54 patients who were not, using an assay which measures the conversion of [alpha-32P]ATP to triphospho(adenylyl 2',5')adenosine. 2,5-A synthetase activity was compared with response to intensive therapy and with duration of maintenance therapy. Seventy-three per cent of patients had measurable amounts of 2,5-A synthetase during the first 6 months of maintenance therapy. This percentage decreased with longer follow-up but not significantly. There was no difference between the magnitude of enzyme induction amongst patients who were in complete remission, partial response or who had no change in disease status following intensive therapy. Peripheral blood T cells were a major source of 2,5-A synthetase activity in patients receiving the cytokine. However, both T and B cells produced the enzyme following exposure to IFN-alpha in vitro. The data show that the level of 2,5-A synthetase in patients with multiple myeloma is not indicative of clinical response to IFN-alpha 2b.


British Journal of Cancer (1995) 72, 1525-1530
© 1995 Stockton Press All rights reserved 0007-0920/95 $12.00

### 2′,5′-Oligoadenylate synthetase levels in patients with multiple myeloma receiving maintenance therapy with interferon α2b do not correlate with clinical response

BC Millar and JBG Bell

*Section of Academic Haematology, The McElwain Laboratories, Institute of Cancer Research, Sutton, Surrey, UK.*

**Summary** In clinical trials with interferon α2b (IFN-α2b) as maintenance therapy for multiple myeloma, the therapeutic benefit is inconclusive. Although the mechanism(s) by which IFN-α2b prolongs remission in some patients is unknown, 2′,5′-oligoadenylate synthetase (2,5-A synthetase) has been used as an objective indicator that IFN-α2b is active *in vivo*. The enzyme was assayed in cytosol preparations of peripheral blood mononuclear cells (MNCs) from 111 patients who were receiving IFN-α2b and 54 patients who were not, using an assay which measures the conversion of [α-$^{32}$P]ATP to triphospho(adenylyl 2′,5′)adenosine. 2,5-A synthetase activity was compared with response to intensive therapy and with duration of maintenance therapy. Seventy-three per cent of patients had measurable amounts of 2,5-A synthetase during the first 6 months of maintenance therapy. This percentage decreased with longer follow-up but not significantly. There was no difference between the magnitude of enzyme induction amongst patients who were in complete remission, partial response or who had no change in disease status following intensive therapy. Peripheral blood T cells were a major source of 2,5-A synthetase activity in patients receiving the cytokine. However, both T and B cells produced the enzyme following exposure to IFN-α *in vitro*. The data show that the level of 2,5-A synthetase in patients with multiple myeloma is not indicative of clinical response to IFN-α2b.

**Keywords:** multiple myeloma; interferon-α2b; 2′,5′-oligoadenylate synthetase

The interferons are a heterogeneous group of proteins that have pleiotropic effects as well as their known anti-viral activity. Interferon α (IFN-α) is the most widely used and is available commercially, from recombinant technology, as IFN-α2a (Roferon), IFN-α2b (Intron-A) and IFN-α2c.

The therapeutic benefit of IFN-α as maintenance therapy for patients with multiple myeloma remains inconclusive. In the first randomised trial, the initial suggestion by Mandelli *et al.* (1990) that IFN-α might produce a survival benefit, following chemotherapy to objective response, was not confirmed with longer follow-up of the same patients (Avvisati *et al.*, 1993). Two other randomised trials produced similar results, although all three studies confirmed a significant prolongation of remission compared with patients who received no further treatment (Ludwig *et al.*, 1991; Westin, 1993). In three other randomised studies, maintenance therapy with IFN-α produced neither prolongation of remission nor a survival benefit (Peest *et al.*, 1990; Bird *et al.*, 1993; Salmon *et al.*, 1994). Results from a non-randomised French study suggest that IFN-α can prolong remission after chemotherapy with autologous bone marrow rescue (ABMR)(Attal *et al.*, 1992). Also in an ongoing study at the Royal Marsden Hospital patients who were randomised to receive IFN-α2b after high-dose melphalan (HDM) and ABMR show a significantly longer duration of remission and survival compared with those who have had no further treatment (Cunningham *et al.*, 1993). However, in both of these studies the benefit of IFN-α was confined to patients who entered complete remission after ABMR. These data suggest that the efficacy of IFN-α in stabilising residual disease is greatest when there has been a substantial reduction of the tumour burden.

The mechanism(s) by which IFN-α stabilises remission in some patients is unknown. It has yet to be determined whether the cytokine acts directly by inhibiting the proliferation of the malignant clone or indirectly by immune modulation. Furthermore, it is not known whether IFN-α remains biologically active when given long term to patients, or whether it is necessary to give IFN-α throughout the duration of remission until relapse. We have shown previously that neutralising antibody production against IFN-α in patients who receive the cytokine as maintenance therapy is transitory and unlikely to account for relapse (Bell *et al.*, 1994). However, recent evidence suggests that some cancer patients who receive IFN-α produce an inhibitor (IIF) which blocks the effects of the recombinant cytokine *in vitro* and is not antibody (Medenica *et al.*, 1994). This raises the possibility that IFN-α may be ineffective in some patients and may be wasteful of an expensive resource.

Treatment of cells with IFN-α results in the induction of 2′-5′ oligoadenylate synthetase (2,5-A synthetase) which catalyses the polymerisation of ATP to triphospho(adenylyl 2′,5′)adenosine (Kerr and Brown, 1978; Baglioni, 1979). This oligomer is required for the activity and stability of ribonuclease L which degrades mRNA and rRNA and consequently inhibits protein synthesis (Nilsen *et al.*, 1981). An increase in the activity of 2,5-A synthetase is a very sensitive indication that cells have been in contact with IFN-α. In patients, induction reaches a maximum approximately 8 h after the administration of the cytokine and elevated levels persist for at least 24 h following cessation of treatment. However, the magnitude of enzyme induction in individual patients shows considerable variation (Merritt *et al.*, 1985). In patients with chronic lymphocytic leukaemia (CLL) receiving IFN-α, the degree of induction of mRNA for 2,5-A synthetase was statistically different between good, intermediate and poor responders, suggesting that measurement of the enzyme might be indicative of clinical response (de Mel *et al.*, 1990). Furthermore, although the exact role of 2,5-A synthetase in the action of IFN-α is unclear, in patients with hairy cell leukaemia and B-cell CLL its induction has been linked directly to the anti-proliferative effect of pentostatin (Ho *et al.*, 1992). In our patients induction and high-dose therapy before maintenance with IFN-α2b, results in a substantial reduction in the tumour burden. Thus, conditions *in vivo* may not be comparable with those in patients with other haematological malignancies who have had no other treatment. Furthermore, since the stem cell in multiple myeloma is unknown it is not possible to determine whether IFN-α2b has a direct effect on the malignant clone, partic-

Correspondence: BC Millar, Section of Academic Haematology, The McElwain Laboratories, Institute of Cancer Research, 15 Cotswold Road, Belmont, Sutton, Surrey, SM2 5NG, UK
Received 6 March 1995; revised 6 July 1995; accepted 12 July 1995

ularly when the number of putative precursors cells are likely to have been substantially reduced. However, because induction of 2,5-A synthetase *in vitro* in phytohaemagglutinin-treated peripheral blood MNC from normal donors and patients with hairy cell leukaemia correlates with the antiproliferative effect of IFN-α2b (Billard *et al.*, 1987), we feel justified in using MNC from our patients as a starting point to investigate the possible contribution of this enzyme.

In this report, we measured 2,5-A synthetase activity in peripheral blood mononuclear cells from patients receiving maintenance therapy with IFN-α2b after HDM followed by ABMR or autologous peripheral blood stem cell rescue (PBSCR). The aims of the experiments were to determine whether there was a significant difference in the magnitude of enzyme induction between patients who have a complete remission, a partial response or no further response to intensive therapy. We also measured the levels of 2,5-A synthetase in patients at different times from the start of maintenance therapy to determine whether there was a decline in activity following prolonged exposure to IFN-α2b. In patients from whom sequential blood samples were available we determined whether loss of enzyme activity is indicative of relapse.

### Materials and methods

### *Clinical samples*

Patients and donors gave informed consent for blood samples to be taken, following approval from the local ethics committee at the Royal Marsden Hospital. Peripheral blood samples were collected by venepuncture from multiple myeloma patients and normal donors at outpatient clinics. Wherever possible, patients were requested to undergo venepuncture at 3-monthly intervals during treatment with IFN-α2b. Amongst patients who were receiving IFN-α2b, blood samples were taken within 24 h of drug administration.

Patients who presented at the Royal Marsden Hospital received one of two conditioning regimens, either CY-VAMP [i.v. infusion of vincristine, 0.4 mg and doxorubicin, 9 mg $m^{-2}$, over 24 h for 4 days with bolus methylprednisolone (1.5 g i.v. or orally daily for 5 days) plus cyclophosphamide (500 mg i.v. bolus on days 1, 8 and 15)] or VERCY-VAMP [vincristine, doxorubicin, methylprednisolone and cyclophosphamide (doses as before) plus verapamil (10 mg i.v. over 24 h for 5 days)] followed by HDM either 200 mg $m^{-2}$ with ABMR or PBSCR or 140 mg $m^{-2}$ alone. Patients were given IFN-α2b (Intron-A, Schering-Plough) at $3 \times 10^6$U $m^{-2}$ subcutaneously three times weekly when the leucocyte count was greater than $3 \times 10^9 l^{-1}$ and platelets were greater than $100 \times 10^9 l^{-1}$.

### *Clinical status*

A complete remission (CR) was defined as the absence of measureable paraprotein and bone marrow infiltration by myeloma cells of $<5\%$. A partial response (PR) was defined as a paraprotein reduced by 50% and improvement in clinical features sustained for longer than 1 month.

### *Preparation of cell extracts*

Mononuclear cells (MNCs) were isolated within 2 h of venepuncture following separation over Ficoll–Hypaque (Boyum, 1968). They were resuspended in RPMI-1640 containing 10% fetal calf serum (FCS) and counted. Aliquots of cell suspensions containing approximately $6 \times 10^6$ MNCs were pelleted at 12 000 g for 30 s. The medium was aspirated from the cells and residual red cell contamination was removed by incubation with Ortho lysis buffer (Ortho Diagnostics Systems, UK) for 10 min at room temperature. The cells were pelleted as before and resuspended in 50–100 μl of lysis buffer [20 mM Hepes, pH 6.9, 5 mM potassium chloride, 5 mM $Mg(OAc)_2$, 1 mM dithiothreitol (DTT) and 0.5% Nonidet P140]. MNCs were subjected to two cycles of freezing and thawing at −80°C followed by ultrasonication for 2 min. Cell debris was removed by centrifugation at 12 000 *g* for 2 min and the supernatants were stored at −80°C until use. This procedure did not result in loss of 2,5-A synthetase activity compared with that found in samples assayed immediately after preparation of cell extracts.

Aliquots of 5 μl of each cell extract were used to determine the protein concentration using a UV spectrophotometer (Shimadzu UV 1201) calibrated at 260 nm.

### *Assay for 2′,5′-oligoadenylate synthetase*

The method used to measure 2,5-A synthetase was similar to that developed by Minks *et al.* (1979). The assay buffer consisted of 0.12 M Mg $(OAc)_2$, 20 mM Hepes/potassium hydroxide buffer, pH 7.4, 5 mM ATP, 1 mM DTT and 10 μg $ml^{-1}$ polyI:polyC. For assay purposes, 4 μl of α-labelled [$^{32}$P]ATP (3000 Ci $M^{-1}$, Amersham International, cat. no. PB 10200) was added to 1 ml of assay buffer at the start of each experiment. An aliquot of 1 μl of the complete buffer was used to determine the total radioactivity in the reaction mixture. This was approximately 100 000 c.p.m. $\mu l^{-1}$.

The assay contained 5 μl of cell extract and 20 μl of assay buffer. Duplicate samples of each cell extract were incubated with gentle agitation at 37°C for 19 h. At this time, oligonucleotides were digested with 2 μl of T-2 ribonuclease (0.1 U; from *Aspergillus oryzae*, Sigma, UK) or snake venom (0.001 U; *Crotalus atrox*, Sigma, UK) for 1 h at 37°C. All extracts were then treated with bacterial alkaline phosphatase (0.1 U; *Escherichia coli*, Sigma Ltd, UK) for 90 min at 37°C. The cell extracts were then heated at 95°C for 2 min and centrifuged at 12 000 *g* for 2 min. Aliquots of 2 μl were spotted onto thin-layer chromatography plates (0.1 mm cellulose MN polyethyleneimine impregnated; Polygram, Macherey-Nagel, Germany) and run with 1 M lithium chloride solvent for 2 h. The chromatograms were autoradiographed overnight (Biomax FilmX-OMAT, Kodak, USA) and the products located on the chromatograms from the developed autoradiographs. Products were cut from the chromatograms and counted in a β-counter in 10 ml of scintillation fluid (Ultima Gold; Packard, USA). Preliminary experiments were done to confirm the stability of the enzyme following freezing and thawing and the nature of the products which were mostly dimers (Minks *et al.*, 1979; Merritt *et al.*, 1985). In addition, MNCs were assayed from a normal volunteer during two separate infectious episodes immediately and after incubation for 24 and 48 h to determine the half-life of 2,5-A synthetase, which was shown to be 24 h (data not shown).

### *Purification of B and T-cells*

The number of T and B cells in MNC were estimated using dual-labelled fluorescence-activated cell analysis (FACS) with monoclonal antibodies conjugated to fluorescene isothiocyanate (FITC) or phycoerythrin (PE) and directed against CD19 and CD3 (Beckton Dickinson, UK,) and CD4, CD8, CD14 and CD45 (Sigma, UK) using an Ortho Cytoron and the software package provided by the manufacturer (Ortho Diagnostics Systems, UK). B and T cells were isolated using antibody coated magnetic beads conjugated to CD19 (B cell) or CD2 (T cell) with a bead to cell ratio of 3:1 and detached from the same by methods described by the manufacturer (Dynal, UK). Viable cells were counted in a haemocytometer using trypan blue exclusion. Isolated B and T cells were incubated at $2 \times 10^6$ $ml^{-1}$ in RPMI-1640 supplemented with 10% FCS, 100 μg $ml^{-1}$ penicillin and 100 U $ml^{-1}$ streptomycin and containing 100 U $ml^{-1}$ IFN-α2b (Intron A) or the same volume of phosphate-buffered saline (PBS). After incubation for 20 h, cells were harvested and processed as above. In experiments in which T cells were isolated from the peripheral blood of patients who were receiving IFN-α2b, cell extracts were prepared immediately after separation from the MNCs, without detachment from magnetic beads.

*Statistical analysis*

Statistical analysis was done using non-parametric tests in a software package.

### Results

Two hundred samples from 111 patients who were receiving IFN-α2b and 87 samples from 54 patients who had not received the cytokine were available for testing.

The data in Table I show that 2,5-A synthetase activity was detected in 36/54 (67%) of patients with multiple myeloma who were not receiving maintenance therapy with IFN-α2b. These included nine patients who had had no previous chemotherapy. The remainder were patients who had had induction therapy alone (11 patients) or induction therapy followed by HDM/ABMR or PBSCR (16 patients). Since enzyme activity in these patients was likely to be due to infection which was documented in some patients after intensive therapy, the data suggest that their response to endogenous IFN was not impaired by intensive therapy. Three of 12 normal donors who were known to have infection at the time of testing also had measurable enzyme activity.

Amongst patients who were having maintenance therapy with IFN-α2b 73% had measurable 2,5-A synthetase activity during the first 6 months of treatment. With longer follow-up this percentage decreased but not significantly. There was considerable variation in the absolute amount of enzyme in individual patients, which showed no correlation with the clinical response to intensive therapy. Although there was a tendency for enzyme activity to decline with longer periods of maintenance therapy this was not statistically significant (Table I).

During the time period over which samples were accrued, peripheral blood from 8/22 patients who remain in CR after HDM/ABMR and who are included in the Royal Marsden Hospital Trial with IFN-α2b as maintenance therapy (Cunningham *et al.*, 1993) was available for testing. Five of these eight patients had measurable levels of 2,5-A synthetase activity 34–72 months after commencing therapy with IFN-α2b (Table II).

Examination of sequential blood samples from individual patients shows that in three patients loss of 2,5-A synthetase activity was associated with relapse (Table III, patients 9, 12 and 14). Two of these patients had had PBSCR (patients nine and 14) and one had had ABMR (patient 12) after HDM. In one of these patients (patient 14) maintenance therapy with IFN-α2b was continued during progressive disease and the enzyme activity increased subsequently. Throughout the duration of maintenance therapy neutralising antibodies to IFN-α2b were not detected. In two patients who failed to show induction of the enzyme, there was no way of identifying whether relapse was imminent (Table III, patients 11 and 13). Both of these patients had had HDM with PBSCR.

When enzyme activity was compared in purified T cells and MNCs from three patients who were receiving IFN-α2b there was substantially more enzyme activity in the T-cell fraction (Table IV). However, both B cells and T cells produced substantial amounts of 2,5-A synthetase following overnight exposure to recombinant IFN-α2b *in vitro* (Table V).

### Discussion

At the Royal Marsden Hospital the majority of newly diagnosed patients with multiple myeloma will receive maintenance therapy with IFN-α2b after intensive therapy with HDM/PBSCR. This is based on the encouraging results from an ongoing study in which more than 50% of patients who entered CR after HDM/ABMR followed by maintenance therapy with the cytokine remained in remission for more than 4 years and have had a significant survival advantage (Cunningham *et al.*, 1993). The mechanism(s) which is involved in mediating these effects are unclear.

In solid tumours, such as melanoma and renal cell carcinoma the anti-tumour effect of IFN-α may be mediated by the immune response, including the activation of NK cells and the enhanced expression of cell-surface markers (e.g. MCH 1 and II: Dorr, 1993). However, there is no evidence that natural killer (NK) (Galvani *et al.*, 1989; Klingemann *et al.*, 1991), lymphokine-activated killer (LAK) or T-cell cytotoxicity (Griffiths and Cawley, 1988) is involved in the anti-tumour response in haematological malignancies.

In an interleukin 6 (IL-6) dependent myeloma cell line IFN-α inhibits proliferation *in vitro* by depriving cells of

**Table II** 2′-5′Oligoadenylate synthetase activity in MNCs from patients in CR receiving IFN-α2b therapy after HDM/ABMR

| *Patient* | *Time of IFN-α2b Treatment (months)* | *Enzyme activity (p mol/µg$^{-1}$ protein h$^{-1}$)* |
|---|---|---|
| 1 | 22 | 4.4 |
| | 35 | 3.02 |
| 2 | 37 | −ve |
| | 43 | −ve |
| 3 | 26 | 11.1 |
| 4 | 58 | 5.08 |
| 5 | 24 | 3.13 |
| 6 | 75 | −ve |
| 7 | 52 | 48.8 |
| 8 | 26 | −ve |

**Table I** Correlation of 2′-5′oligoadenylate synthetase activity in MNCs from multiple myeloma patients before and during maintenance therapy with IFN-α2b with clinical status

| *Time of treatment* | *Total number of patients* | *Patients with detectable enzyme activity No.* | *(%)* | *Enzyme activity in pmol µg$^{-1}$ protein h$^{-1}$ Median (range)* | *Clinical status (no. of patients)* | *Enzyme activity in pmol µg$^{-1}$ protein h$^{-1}$ Median (range)* |
|---|---|---|---|---|---|---|
| Pretreatment | 54 | 36 | (67) | 11.0 (2.02–148) | | |
| 0–6 months | 52 | 38 | (73) | 13.7 (2–166) | CR (12) | 11.20 (2.6–100) |
| | | | | | PR (14) | 15.25 (2.0–166) |
| | | | | | NC/PD (12) | 13.10 (3.2–58.3) |
| 7–12 months | 22 | 11 | (50) | 8.78 (2.27–80.6) | CR (5) | 8.78 (2.27–28) |
| | | | | | PR (3) | 36.60 (3.4–80.6) |
| | | | | | NC/PD (3) | 7.10 (4.3–33.9) |
| >12 months | 37 | 24 | (65) | 4.89 (2.1–48.8) | CR (9) | 5.08 (3.02–48.8) |
| | | | | | PR (6) | 7.78 (2.8–16.7) |
| | | | | | NC/PD (9) | 4.68 (2.1–27.8) |
| Normal | 12 | 3 | (25) | 4.5 (3.6–7.2) | | |

CR, complete remission; PR, partial response; NC, no change or plateau phase; PD, progressive disease.

**Table III** 2'5'Oligoadenylate synthetase activity in MNC taken sequentially from multiple myeloma patients during maintenance therapy with IFN-α2b

| *Patient* | *Clinical status* | *Time of interferon-α2b treatment (months)* | *Enzyme activity (pmol μg⁻¹ protein h⁻¹)* |
|---|---|---|---|
| | CR | 7 | 2.27 |
| 9 | CR | 12 | 18.5 |
| | CR | 18 | −ve |
| | PD | 20 | −ve |
| | CR | 1 | 17.0 |
| | CR | 5 | 7.95 |
| 10 | CR | 8 | 10.0 |
| | CR | 9 | 28.0 |
| | CR | 13 | 11.0 |
| 11 | CR | 5 | −ve |
| | CR | 8 | −ve |
| | | Died at 14 | |
| | PR | 6 | 20.7 |
| | PR | 9 | 37.8 |
| 12 | PR | 12 | 35.4 |
| | PR | 13 | 16.7 |
| | PR | 15 | 1.6 |
| | PD | PD at 20 | −ve |
| | NC | 1 | −ve |
| 13 | NC | 2 | −ve |
| | NC | 7 | −ve |
| | NC | 12 | −ve |
| | PR | Pretreatment | 4.2 |
| | CR | 1 | 16.0 |
| | CR | 6 | 1.2 |
| 14 | CR | 9 | 0.34 |
| | PD | 10 | 16.3 |
| | NC | 11 | 33.9 |
| | NC | 12 | 6.2 |

CR, complete remission; PR, partial response; NC, no change or plateau phase; PD, progressive disease.

**Table IV** Comparison of 2',-5'-oligoadenylate synthetase activity in MNCs and T cells in multiple myeloma patients receiving IFN-α2b therapy

| *Patient* | *Enzyme activity (pmol μg⁻¹ protein h⁻¹)* MNCs | *T cells* |
|---|---|---|
| 15 | 3.25 | 55.8 |
| 16 | 11.1 | 126.3 |
| 17 | 3.8 | 25.0 |

**Table V** 2',-5'-Oligoadenylate synthetase activity in isolated B and T cells from multiple myeloma patients not receiving IFN-α2b following exposure to 100 U ml⁻¹ IFN-α2b *in vitro*

| *Patient* | *Cell type* | *Enzyme activity (pmol μg⁻¹ protein h)* |
|---|---|---|
| 18 | B cells | 58.9 |
| 19 | B cells | 156.8 |
| 20 | T cells | 7.3 |
| 21 | T cells | 14.7 |
| 22 | T cells | 68.1 |

functional IL-6 · receptors, thereby interrupting an essential autocrine loop (Schwabe *et al.*, 1994). Other workers have suggested that a similar mechanism may be involved in the anti-proliferative effect of IFN-α in patients with hairy cell leukaemia and B-cell leukaemia and that it may be mediated by 2,5-A synthetase (Heslop *et al.*, 1991).

In this study, there was no correlation between disease status after intensive therapy for multiple myeloma and our ability to measure 2,5-A synthetase. The finding that the enzyme was not measurable in a significant number of patients suggests that resistance to induction of this protein by IFN-α2b can develop early during treatment. In CML, resistance to IFN-α has been ascribed to post-translational modification of the enzyme since there was no consistent defect in the expression of mRNA transcripts for 2,5-A synthetase in patients who were resistant or sensitive to the cytokine (Talpaz *et al.*, 1992).

Among patients who had measurable levels of 2,5-A synthetase there was considerable inter-patient variation, in agreement with earlier studies (Merritt *et al.*, 1985) but there was no correlation between the magnitude of the enzyme activity and response to intensive therapy. Some of this variability is likely to be due to the distribution of cell types within individual samples and the contribution that each cell type makes to the total enzyme activity in the MNC fraction. In our patients, T lymphocytes were a major source of the enzyme, however it is likely that both B and T cells contribute to enzyme activity *in vivo* since both cell types showed enzyme induction after exposure *in vitro* to IFN-α2b.

The loss of enzyme activity at progressive disease in three patients suggests that the mechanism(s) which maintains multiple myeloma in a non-proliferative state can be evaded in the absence of 2,5-A synthetase. However, it is not proof that 2,5-A synthetase is a prerequisite for maintaining tumour homeostasis. Once tumour cell proliferation had been initiated *in vivo*, subsequent recovery of the enzyme as in the patient who continued to receive IFN-α2b during relapse, was not accompanied by inhibition of tumour cell proliferation. Furthermore, 2,5-A synthetase was not detected in three patients who remain in long-term remission but was found in nine patients at presentation. In a study of seven patients receiving IFN-α for hairy cell leukaemia, one of two non-responders had similar levels of 2,5-A synthetase to those found in patients who responded to the cytokine (Billard *et al.*, 1988). Collectively this report and our data suggest that other mechanisms which do not involve 2,5-A synthetase may be involved in the anti-tumour effect of IFN-α2b.

In the study of IFN-α as maintenance therapy for multiple myeloma by the South West Oncology Group, although

IFN-α2b alone was ineffective, addition of glucocorticoids improved the clinical outcome (Salmon *et al.*, 1994). The authors proposed that long-term glucocorticoid administration in multiple myeloma during both remission induction and maintenance may result in the best survival overall. At the Royal Marsden Hospital all patients who have had HDM/ABMR received methylprednisolone 1 g $m^{-2}$ for 5 days immediately after HDM and before the start of maintenance therapy (Cunningham *et al.*, 1993). Whether the continued CR of three out of eight of these patients, despite the absence of 2,5-A synthetase, was determined by the glucocorticoid treatment *per se* or augmented an antiproliferative effect which was independent of the enzyme cannot be resolved. In the current treatment protocol patients who have had HDM/PBSCR have not had methylprednisolone after HDM. The finding that two patients who have relapsed from CR after HDM/PBSCR at 20 months (patient 9) and 10 months (patient 14) following a decline in 2,5-A synthetase while receiving IFN-α2b may be relevant to this proposition. Whether administration of methylprednisolone after HDM/ABMR has been a determining factor in the prolongation of remission and/or survival benefit in patients who achieved CR and were given IFN-α2b subsequently may become apparent as patients are followed who have PBSCR.

In conclusion, the findings that some patients did not produce 2,5-A synthetase while receiving the cytokine suggests either that IFN-α2b was inactive in these patients or that induction of this enzyme is irrelevant to the suppression of the malignant clone by IFN-α2b. In other haematological malignancies the correlation between 2,5-A synthetase levels and clinical response occurs in patients who receive IFN-α2b as sole treatment (de Mel *et al.*, 1990; Ho *et al.*, 1992) suggesting that enzyme levels reflect an antiproliferative effect. However, this may not be analogous to the conditions in our patients who have had intensive therapy before receiving the cytokine and in whom IFN-α2b is given to prolong the response to HDM rather than illicit tumour cell kill. Although the mechanism(s) involved in tumour growth inhibition remains elusive the data suggest that there is a critical balance between the tumour mass and its proliferative state which must be achieved to enable IFN-α2b to maintain homeostasis. Further studies may determine whether restoration of normal haemopoiesis, which may effect tumour homeostasis, occurs independently of the induction of 2,5-A synthetase in multiple myeloma patients receiving IFN-α2b as maintenance therapy. Such studies may result in a more effective and economical use of an expensive resource.

**Acknowledgements**
We thank the Cancer Research Campaign for funding this work, Dr RL Powles and the staff and patients at the Royal Marsden Hospital, Sutton, Surrey for clinical samples and Miss S Fernando for technical help.

**References**

ATTAL M, HUGUET F, SCHLAIFER D, PAYEN C, LAROCHE M, FOURNIE B, MAZIERES B, PRIS J AND LAURENT G. (1992). Intensive combined therapy for previously untreated aggressive myeloma. *Blood*, **79**, 1130–1136.

AVVISATI G, BOCCADORO M AND PETRUCCI MT. (1993). Interferon alpha as maintenance treatment in multiple myeloma: the Italian experience. Proceedings of the Fourth International Workshop on Multiple Myeloma, 2–5 October 1993, Rochester, USA, 87–88.

BAGLIONI C. (1979). Interferon-induced enzymatic activities and their role in the antiviral state. *Cell*, **17**, 255–264.

BELL JBG, BARFOOT R, IVESON T, POWLES RP AND MILLAR BC. (1994). Neutralising antibodies in patients with multiple myeloma receiving maintenance therapy with interferon α2b. *Br. J. Cancer*, **70**, 646–651.

BILLARD C, FERBUS D, KOLB JP, ROSA F, PERROT JY, MERLIN G, JANIAURD P, RAYNAUD N, THANG MN AND FELLOUS M. (1987). Qualitative differences in effects of recombinant alpha-, beta-, and gamma- interferon on human peripheral blood leukocytes in vitro. *Ann. Inst. Pasteur Immunol.*, **137C**, 259–279.

BILLARD C, FERBUS D, SIGAUX F, CASTIAGNE S, DEGO L, FLANDRIN G AND FALCOFF E. (1988). Action of interferon-alpha on hairy cell leukaemia: expression of specific receptors and (2'5')oligo(A) synthetase in tumour cells from sensitive and resistant patients. *Leuk. Res.*, **12**, 11–18.

BIRD JM, SAMSON D AND NEWLAND A. (1993). A randomized study comparing VAD with current INF with VAD following maintenance INF in newly diagnosed myeloma. Proceedings of the Fourth International Workshop on Multiple Myeloma, 2–5 October 1993, Rochester, USA, p 130.

BOYUM A. (1968). Separation of leucocytes from blood and bone marrow. *Scand. J. Clin. Lab. Invest.*, **21**, 1–6.

CUNNINGHAM D, POWLES R, MALPAS JS, MILAN S, MELDRUM M, VINER C, MONTES A, HICKISH T, NICOLSON M, JOHNSON P, MANSI J, TRELEAVEN J, RAYMOND J AND GORE ME. (1993). A randomised trial of maintenance therapy with Intron A following high dose melphalan and ABMT in myeloma. *Br. J. Cancer*, **67**, (suppl. XX), 30.

DE MEL WC, HOFFBRAND AV, GILES FJ, GOLDSTONE AH, MEHTA AB AND GANESHAGURU K. (1990). Alpha interferon therapy for haematological malignancies; correlation between in vivo induction of the 2',5'oligoadenylate system and clinical response. *Br. J. Haematol.*, **74**, 452–456.

DORR RT. (1993). Interferon-alpha in malignant and viral diseases. A review. *Drugs*, **45**, 177–211.

GALVANI DW, OWENS W, NETHERSELL ABW AND CAWLEY JC. (1989). The beneficial effects of α-inferferon in CGL are probably not due to NK cells. *Br. J. Haematol.*, **71**, 233–237.

GRIFFITHS SD AND CAWLEY JC. (1988). α Interferon and LAK cell activity in hairy-cell leukemia. *Leukemia*, **2**, 377–381.

HESLOP HE, BRENNER MK, GANESHAGURU K AND HOFFBRAND AV. (1991). Possible mechanism of action of interferon alpha in chronic B-cell malignancies. *Br. J. Haematol.*, **79** (suppl. 1), 14–16.

HO AD, KLOTZBUCHER A, GROSS A, DIETZ G, NESTAN J, JAKOBSEN H AND HUNSTEIN W. (1992). Induction of intracellular and plasma 2',5'-oligoadenylate synthetase by pentostatin. *Leukemia*, **6**, 209–214.

KERR IM AND BROWN RE. (1978). pppA2'p5'A2'p5'A: An inhibitor of protein synthesis synthesized with an enzyme fraction from interferon-treated cells. *Proc. Natl Acad. Sci. USA*, **75**, 256–260.

KLINGEMANN HG, GRIGG AP, BOYD KW, BARNETT MJ, EAVES AC, REECE DE, SHEPHERD JD AND PHILLIPS GL. (1991). Treatment with recombinant interferon (α-2b) early after bone marrow transplantation in patients at high risk for relapse. *Blood*, **78**, 3306–3311.

LUDWIG H, COHEN AM, HUBER H, NACHBAUR D, JUNGI WF, SENN H, GUNCZLER P, SCHULLER J, ECKHARDT S, SEEWANN HL, CAVALLI F, FRITZ E AND MICKSCHE M. (1991). Interferon α-2b with VMCP compared to VMCP alone for induction and interferon α-2b compared to controls for remission maintenance in multiple myeloma: interim results. *Eur. J. Cancer*, **27** (suppl. 4), S40-S45.

MANDELLI F, AVVISATA G AND AMARDORI S. (1990). Maintenance with recombinant interferon alpha 2b in patients with multiple myeloma responsive to conventional induction therapy. *New Engl. J. Med.*, **322**, 1430–1434.

MEDENICA R, MUKERJEE S, HUENTEMANN S AND HUSCHART T. (1994). Interferon inhibitor factor in malignant diseases (abstract 493). *Exp. Hematol.*, **22**, 809.

MERRITT JA, BORDEN EC AND BALL LA. (1985). Measurement of 2',5'-oligoadenylate synthetase in patients receiving interferon-α. *J. Interferon Res.*, **5**, 191–198.

MINKS MA, BENVIN S, MARONEY PA AND BAGLIONI C. (1979). Synthesis of 2'5'-oligo(A) in extracts of interferon-treated HeLa cells. *J. Biol. Chem.*, **254**, 5058–5064.

NILSEN TW, WOOD DL AND BAGLIONI C. (1981). 2',5'-Oligo(A)-activated endoribonuclease. Tissue distribution and characterization with a binding assay. *J. Biol. Chem.*, **256**, 10751–10754.

PEEST D, DEICHER D AND COLDEWEY R. (1990). Melphalan and prednisolone (MP) versus vincristine, BCNU, adriamycin, melphalan and dexamethasone (VBAMDex) induction chemotherapy and interferon maintenance treatment in multiple myeloma. Current results of a multicentre trial. *Onkologie*, **13**, 458–460.

SALMON SE, CROWLEY JJ, GROGAN TM, FINLEY P, PUGH RP AND BARLOGIE B. (1994). Combination chemotherapy, glucocorticoids and interferon α in the treatment of multiple myeloma: A Southwest Oncology Group study. *J. Clin. Oncol.*, **12,** 2405–2414.

SCHWABE M, BRINI AT, BOSCO MC, RUBBOLI F, EGAWA M, ZHAO J, PRINCLER GL AND KING HF. (1994). Disruption by interferon α of an autocrine interleukin-6 growth loop in IL6-dependent U266 myeloma cells by homologous and heterologous down-regulation of the IL-6 receptor alpha- and beta-chains. *J. Clin. Invest.*, **94,** 2317–2325.

TALPAZ M, CHERNAJOVSKY Y, WORDEN KT, WETZLER M, KANTARJIAN H, GUTTERMAN JU AND KURZROCK R. (1992). Interferon-stimulated genes in interferon-sensitive and -resistant chronic myelogenous leukemia patients. *Cancer Res.*, **52,** 1087–1090.

WESTIN J. (1993). Alpha interferon for maintenance therapy in multiple myeloma. Proceedings of the Fourth International Workshop on Multiple Myeloma, 2–5 October 1993, Rochester, USA, pp. 89–90.